# Using Upper Extremity Skin Temperatures to Assess Thermal Comfort in Office Buildings in Changsha, China

**DOI:** 10.3390/ijerph14101092

**Published:** 2017-09-21

**Authors:** Zhibin Wu, Nianping Li, Haijiao Cui, Jinqing Peng, Haowen Chen, Penglong Liu

**Affiliations:** College of Civil Engineering, Hunan University, Changsha 410081, China; wuzhibin@hnu.edu.cn (Z.W.); cuihaijiao@hnu.edu.cn (H.C.); Jallenpeng@gmail.com (J.P.); chenhaowen@hnu.edu.cn (H.C.); liupenglong@hnu.edu.cn (P.L.)

**Keywords:** thermal comfort, thermal sensation, thermal acceptability, thermal perception, upper extremity skin temperatures

## Abstract

Existing thermal comfort field studies are mainly focused on the relationship between the indoor physical environment and the thermal comfort. In numerous chamber experiments, physiological parameters were adopted to assess thermal comfort, but the experiments’ conclusions may not represent a realistic thermal environment due to the highly controlled thermal environment and few occupants. This paper focuses on determining the relationships between upper extremity skin temperatures (i.e., finger, wrist, hand and forearm) and the indoor thermal comfort. Also, the applicability of predicting thermal comfort by using upper extremity skin temperatures was explored. Field studies were performed in office buildings equipped with split air-conditioning (SAC) located in the hot summer and cold winter (HSCW) climate zone of China during the summer of 2016. Psychological responses of occupants were recorded and physical and physiological factors were measured simultaneously. Standard effective temperature (SET*) was used to incorporate the effect of humidity and air velocity on thermal comfort. The results indicate that upper extremity skin temperatures are good indicators for predicting thermal sensation, and could be used to assess the thermal comfort in terms of physiological mechanism. In addition, the neutral temperature was 24.7 °C and the upper limit for 80% acceptability was 28.2 °C in SET*.

## 1. Introduction

Thermal comfort is defined as “the condition of mind that expresses satisfaction with the thermal environment” [1], which is a major condition in indoor environment and has received increasing attention [2,3,4,5]. There are two main models for evaluating thermal comfort: the heat balance model [6,7] and the adaptive model [8,9]. The heat balance model is based on climate chamber experiments while the adaptive model is derived from field studies. In the 20th century, Gagge [10,11,12] started a series of studies on human heat balance models and proposed the standard effective temperature (SET*) concept. Fanger [6] developed the predicted mean vote (PMV) model. Adaptive approaches focus on analyzing the real thermal environment, which incorporate the effects of physical, climatic, adaptive, social and cultural factors. De Dear [9] summarized that the adaptive adjustments included the behavior, physiological adaptation, and psychological adaptation. As thermal comfort is subjective and multi-factor dependent, it differs depending on individual preference, clothing, activity level, etc. [13]. The two models can explain the relationship between subjective thermal sensation and thermal environment, but they cannot explain why and how a thermal environment influences the thermal sensation.

The skin, as the sensory organ representing the physiological interface with our surrounding environment, was adopted by many researchers as a thermal comfort indicator. Thermoreceptors were found peripherally in the body (i.e., in the skin) [14], and the thermoreception process allowed humans to effectively regulate autonomic and behavioral thermoregulatory responses [15,16,17]. Variations in the thermal properties of the skin resulting from changes in the thermal environment represent the first step in the biological processes, which result in what we consciously experience as skin thermal sensation. It is now recognized that skin temperature is a physiological factor that is related to the human thermal comfort.

The upper extremity skin, namely the glabrous skin, has many arteriovenous anastomose valves that control vasoconstriction and vasodilatation [18,19]. The number of thermoreceptors in the finger and hand is greater than those in other body skin areas. The upper extremities play an important role in human thermoregulation through vasoconstriction and vasodilatation, which makes them become the most sensitive indicator of thermal sensation. In cool or cold thermal environments, thermoregulation takes place by vasoconstriction and cold upper extremities mean heat will be conserved. In a warm or hot thermal environment, thermoregulation takes place initially by means of vasodilatation and subsequently by sweating [20]. Warm upper extremities indicate that more heat is dissipating to the environment.

Many researchers have considered skin temperature for evaluating personal thermal sensations. The mean skin temperature has been used to assess thermal sensation in various studies [21,22,23,24,25,26]. However, research exploring the relationships between upper extremity skin temperatures and thermal sensation is limited. Humphreys [27] explored the relationship between the finger temperature and overall thermal sensation and concluded that finger temperature was useful in explaining the variation in sensation votes in building surveys. The correlation coefficient was improved from 0.31 to 0.43 by adding finger temperature to globe temperature in fitting thermal sensation. Wang [28] explored how upper extremity skin temperatures correlated with thermal comfort by repeated surveys in a climate chamber. The results indicated that finger temperature (30 °C) and finger-forearm temperature gradient (0 °C) are significant thresholds for overall thermal sensation. Nakayama [29] developed a thermal comfort prediction algorithm based on the finger skin temperature and used the algorithm to control a household air conditioner. Sim [30] invented a wrist band that monitored skin temperatures from the wrist and the fingertip and proposed thermal sensation estimation technology based on wrist skin temperature. However, almost all above mentioned studies were conducted in climate chambers, where the thermal environment was highly controlled to several specific thermal conditions and the number of occupants was small. Few comprehensive studies were conducted to determine the relationships between upper extremity skin temperatures and thermal comfort except using the finger. Whether the existing climate chamber theories are suitable for the actual thermal environment is unknown.

The aim of present study was to explore whether upper extremity skin temperatures could predict the thermal comfort in an office-based environment. The hypothesis is that upper extremity skin temperatures are good indicators for predicting thermal sensation, and could be used to assess the thermal comfort in terms of physiological mechanism. In this research, the relationships between thermal comfort and upper extremity skin temperatures were developed in an actual thermal environment. Moreover, the thermal environment of the investigated office buildings were also analyzed. The neutral and acceptable thermal conditions were identified. The analyzed dataset was obtained from the thermal comfort field studies, which were conducted in office buildings located in Changsha, a typical city in the hot summer and cold winter (HSCW) climate zone of China. The field studies consisted of measuring the thermal environment and interviewing the occupants simultaneously. The upper extremity skin temperatures were also measured. Furthermore, the relationships between thermal comfort and upper extremity skin temperatures were assessed by determination coefficient.

## 2. Research Methods 

### 2.1. Time, Climate and Buildings 

Changsha, a typical city in the HSCW climate zone of China, is located at latitude 28°41′ N and longitude 114°15′ E. The HSCW climate zone has an Asian hot monsoon climate, with long hot-humid summers and long cold-humid winters; the city has a humid subtropical climate in terms of the Köppen-Geiger climate classification. The hottest monthly average outdoor air temperature ranges from 25 °C to 30 °C, while the coldest monthly average outdoor air temperature ranges from 0 °C to 10 °C.

**Figure 1:**
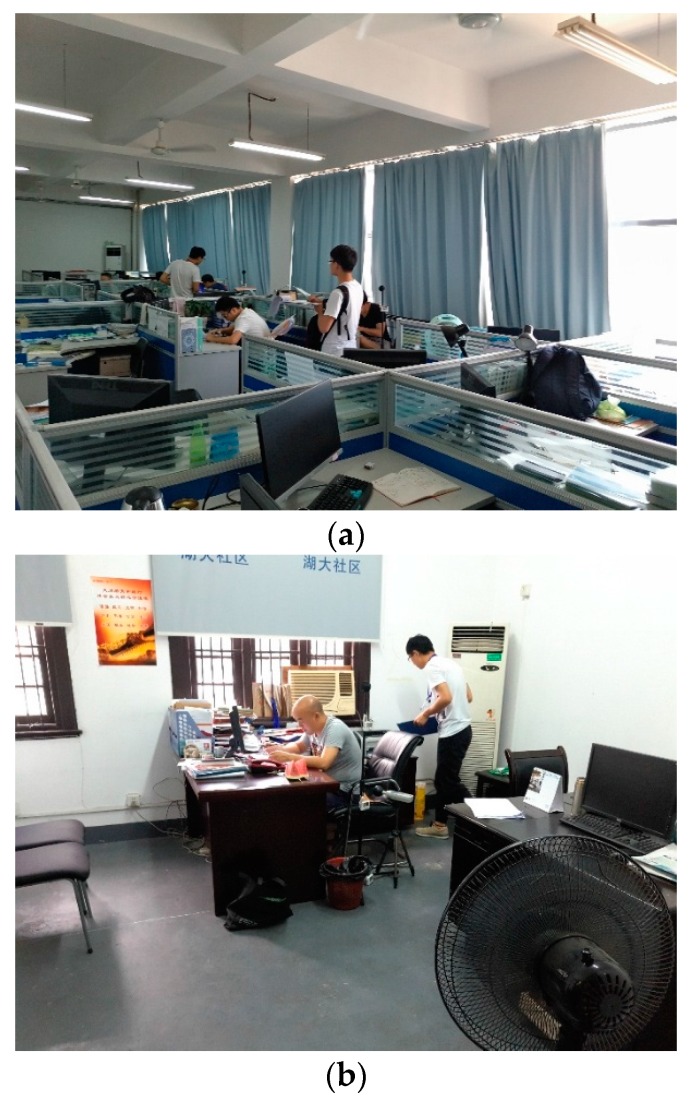
Typical office rooms: (**a**) Open-concept office room (**b**) Private office room.

Thermal comfort field studies were carried out from July to September 2016. During this period, the daily average outdoor temperature in Changsha ranged from 20.9 °C to 32.5 °C (SD = 2.5 °C) while the daily average outdoor relative humidity ranged from 58 to 99% (SD = 11%). The office buildings, located on the campus of Hunan University, were selected as the target buildings for the field studies. The buildings were medium and low in height. Figure 1 shows the typical office rooms, consisting of private and open-concept office rooms. The office buildings were almost all built in last century, with masonry or steel-framed concrete structures, aluminum alloy or wood windows and single-glazed windows. All the office buildings were equipped with split air-conditioners. The windows and doors of buildings could be easily opened or closed by the occupants. Most buildings have other forms of personal comfort systems, such as ceiling fans and desk fans. The main thermal response behaviors of the occupants in the office buildings were adjusting clothing, opening windows and doors, using split air-conditioners and electrical fans.

### 2.2. The Investigated Occupants and Sample Size

A cross-section design [31] was used in this research. Each occupant participated once during the field studies. In total 430 occupants, including 298 males and 132 females, returned valid questionnaires. They were teachers, students and administrators. All occupants were healthy non-smokers who were not taking prescription medication. Their ages ranged from 20 to 53 and the average age was 26. The anthropometric information of the occupants is listed in Table 1.

### 2.3. Physiological Measurements

**Figure 2:**
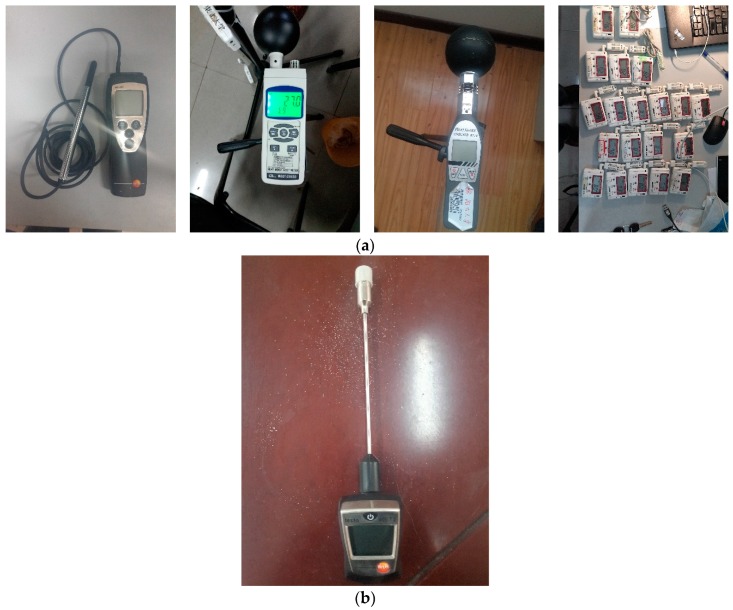
Instruments: (**a**) Physical instruments (**b**) Physiological instruments.

Upper extremity skin temperatures were measured after the subjective survey, using a 905-T2 surface temperature meter (Testo, Lenzkirch, Germany, Figure 2).

In detail, the upper extremity skin temperatures were measured at four upper local skin parts: the positive side of the 3rd finger of the left hand, dorsal side of the left hand, wrist of the left hand and dorsal side of the left forearm. The accuracies of related instruments and detail information are presented in Table 2.

### 2.4. Physical Measurements

**Figure 3:**
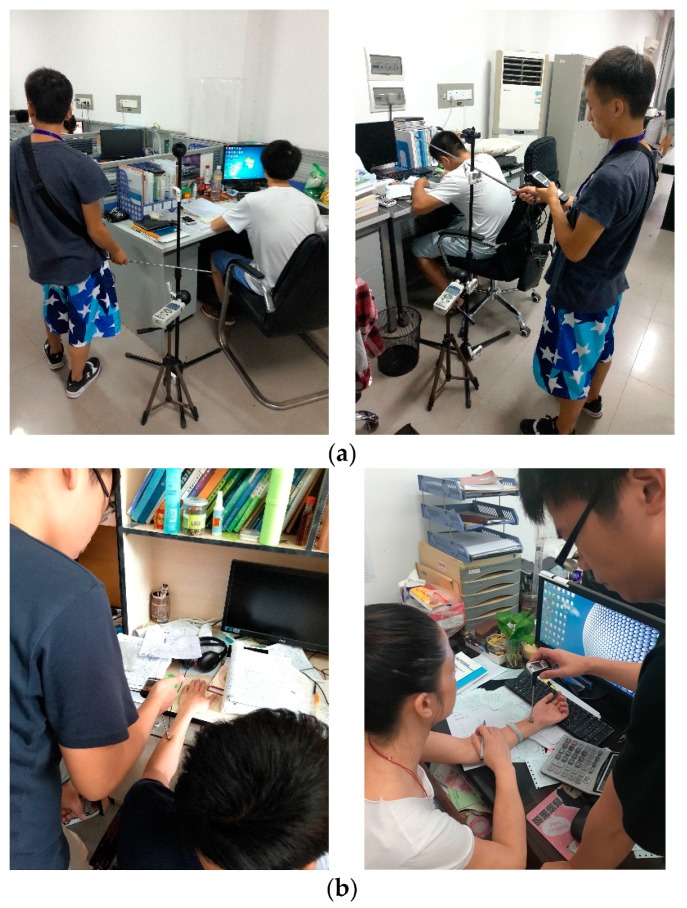
Physical and physiological measurements: (**a**) Physical measurements (**b**) Physiological measurements.

Indoor physical measurements and the subjective survey were conducted simultaneously. According to ASHRAE Standard 55-2013 [1], air temperature (*T_a_*), relative humidity (*RH*), globe temperature (*T_g_*) and air speed (*V_a_*) were measured by laboratory-grade instruments. All of them were close to the participants (within 0.3 m, Figure 3).

The instruments and the corresponding accuracies are listed in Table 2. Ambient temperature and relative humidity were measured using three combined temperature/humidity instruments (TR-72U, T&D Corporation, Tokyo, Japan), which were attached to a tripod, hanging next to the subject at 0.1 m, 0.6 m and 1.1 m heights (from the ground). The 0.6 m and 1.1 m are representative of the height of a human body sitting and standing, respectively. Two 40 mm black spheres were used to measure the globe temperature at 0.6 m height. At the same time, considering the differences in air velocity at different heights, the present study referred the ASHRAE Standard 55-2013 [1] and measured the air velocity by a Testo 425 hot-wire anemometer at 0.1 m, 0.6 m and 1.1 m heights (Figure 3). The corresponding average values were used for further analysis of the environmental variables. Mean radiant temperature (*T_r_*) and operative temperature (*T_o_*) were calculated based on the equations provided by ISO 7726 [33]. Predicted mean vote (PMV) was calculated by using equations in ASHRAE Standard 55-2013 [1]. Considering the hot and humid environment in the field studies, SET* was determined by using the two-node model [10,34]. The method was consistent with ASHRAE Standard 55-2013 [1] and ASHRAE Handbook-Fundamentals 2009 [35]. For example, the detailed equation of calculating *T_r_* was given by:

*T_r_* = [(*t_g_* + 273)^4^ + 2.5 × 10^8^ × *v_a_*^0.6^(*t_g_* − *t_a_*)]^1/4^ − 273, (1)

Based on the propagation of error theory, the error of *T_r_* could be determined as follows:

(2) $$T_{r}=\frac{\partial T_{r}}{\partial t_{g}}\Delta t_{g}+\frac{\partial T_{r}}{\partial v_{a}}\Delta v_{a}+\frac{\partial T_{r}}{\partial t_{a}}\Delta t_{a}$$

where Δ*T_r_*, Δ*t_g_*, Δ*v_a_* and Δ*t_a_* are the errors of *T_r_*, *t_g_*, *v_a_* and *t_a_*, respectively. According to the accuracies of the instruments, the values of Δ*t_g_*, Δ*v_a_* and Δ*t_a_* were 0.6 °C, 0.03 m/s and 0.3 °C, respectively. Substituting the measured values of *t_g_*, *v_a_* and *t_a_*, the mean error of *T_r_* could be obtained as 1.30 °C (SD = 0.32 °C).

### 2.5. Thermal Comfort Survey

**Figure 4:**
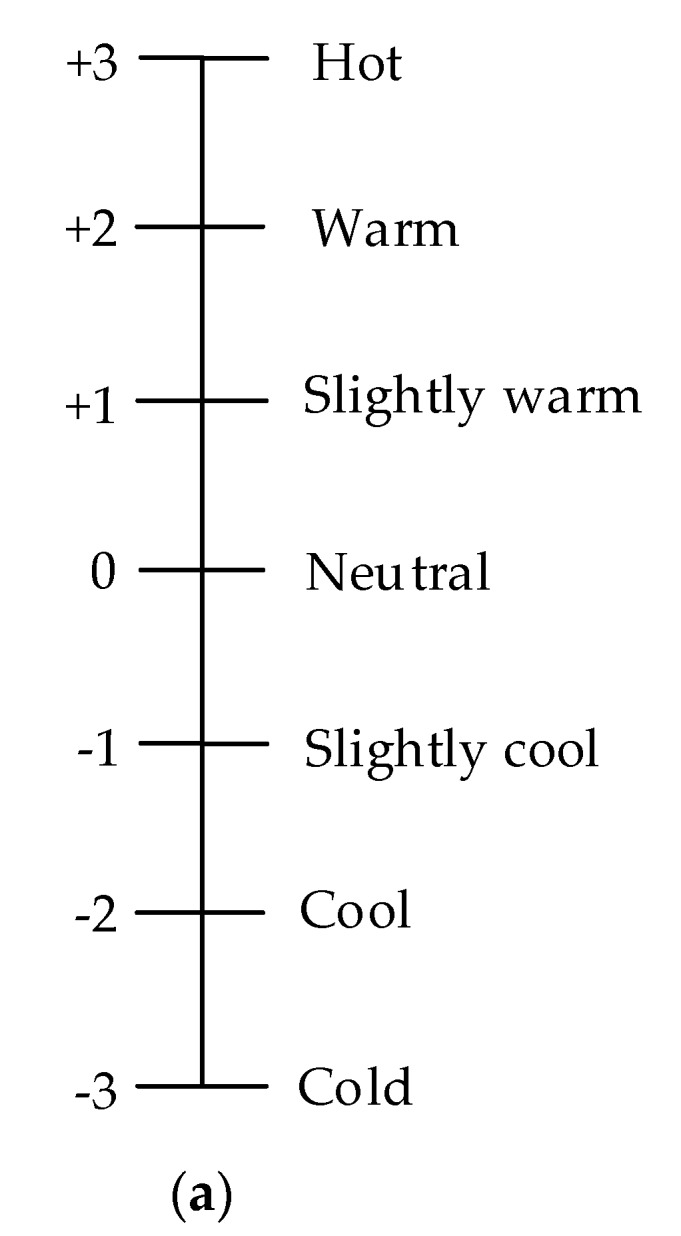

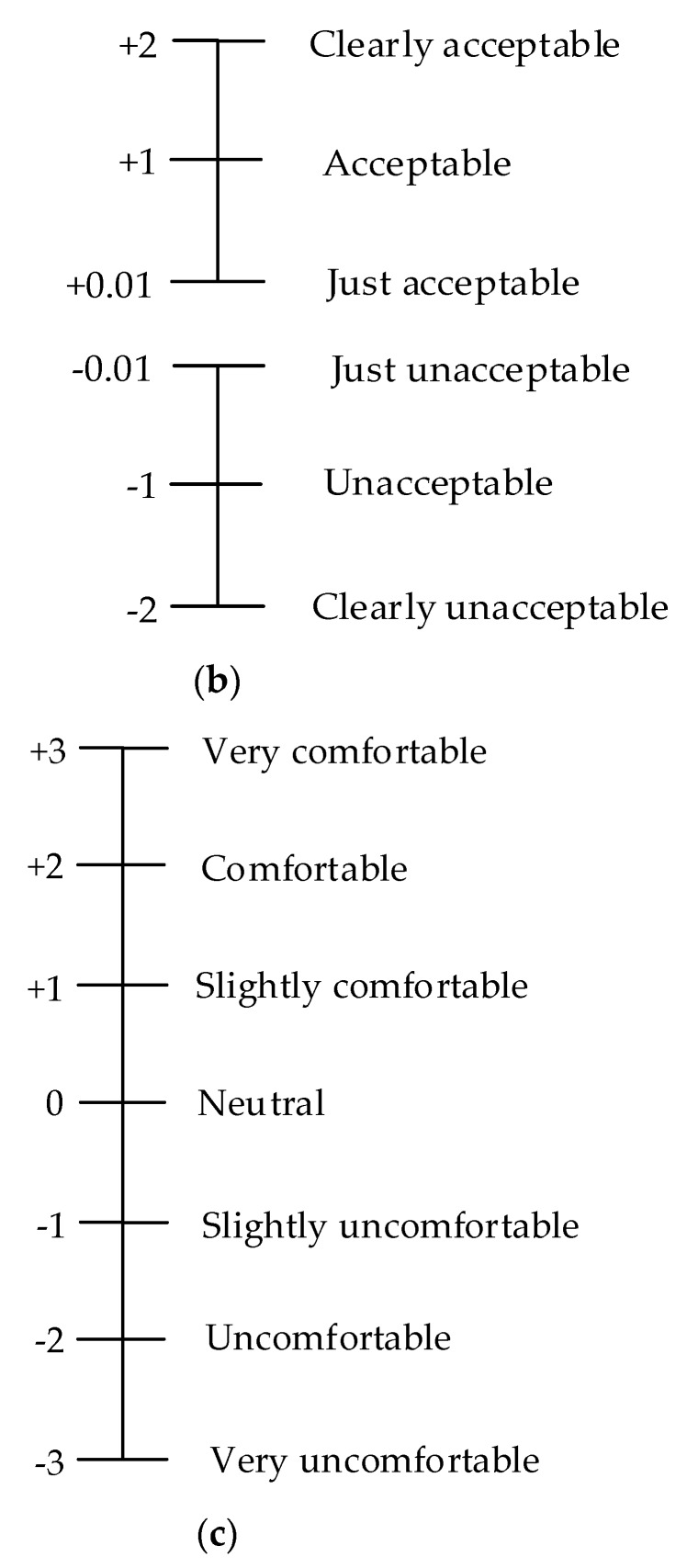
Rating scales: (**a**) Thermal sensation (**b**) Thermal acceptability (**c**) Thermal comfort.

The survey was conducted from 9:00–11:00 am and 15:00–17:00 pm. Questionnaires, which were written in Chinese and presented in paper form were designed to quantify occupants’ personal information and thermal perceptions. The Chinese descriptions of the scales from the Chinese national standard GB/T 18977 (AQSIQ, 2003) [36] were used [37]. The questionnaires included three parts. The first part was a background survey, seeking information such as gender, height, weight, age, the subjects’ activity level and length of residence in Changsha. The second part was the subjective thermal perception in terms of air temperature, relative humidity and air velocity, including thermal sensation, comfort, preferences and acceptability (Figure 4). The final part was concerned with thermal behaviors, such as opening windows or doors, drinking cool drinks and changing clothes. The clothing insulation checklist was done by researchers through consulting the occupants. Individual clothing articles indicated in the responses were quantified in ‘clo’ units based on ASHRAE Standard 55-2013 [1], and the overall clo value for each subject’s entire clothing ensemble was then determined by summing each value of each article and the contribution given by a normal chair (0.1 clo). Similar to the research conducted by Zhang [31], the acceptable percentage was determined as percentage of samples with positive thermal acceptability votes.

### 2.6. Statistic Analysis

The sex differences in upper extremity skin temperatures were tested by Mann-Whitney test. Also, the differences of upper extremity skin temperatures between two nearby thermal sensation groups were assessed by Mann-Whitney test. Kruskal-Wallis test was used to determine whether thermal sensation had significant impacts on the upper extremity skin temperatures. All regression correlations were performed with a one-way ANOVA test. All the statistical analyses were made with the GraphPad Prism v7 software (Graphpad Software, San Diego, CA, USA), and all the differences were accepted as significant at the 0.05 level.

## 3. Results

### 3.1. Thermal Environment

In this section, the indoor thermal environment is analyzed. The distributions of the indoor thermal variables are shown in Table 3. The indoor mean air temperature was 26.8 °C. The relative humidity ranged from 30 to 90%, and the average value was 60%. The air velocity ranged from 0 to 1.3 m/s, and the average value was 0.2 m/s.

### 3.2. Relationship between Thermal Sensation and Thermal Environment

**Figure 5:**
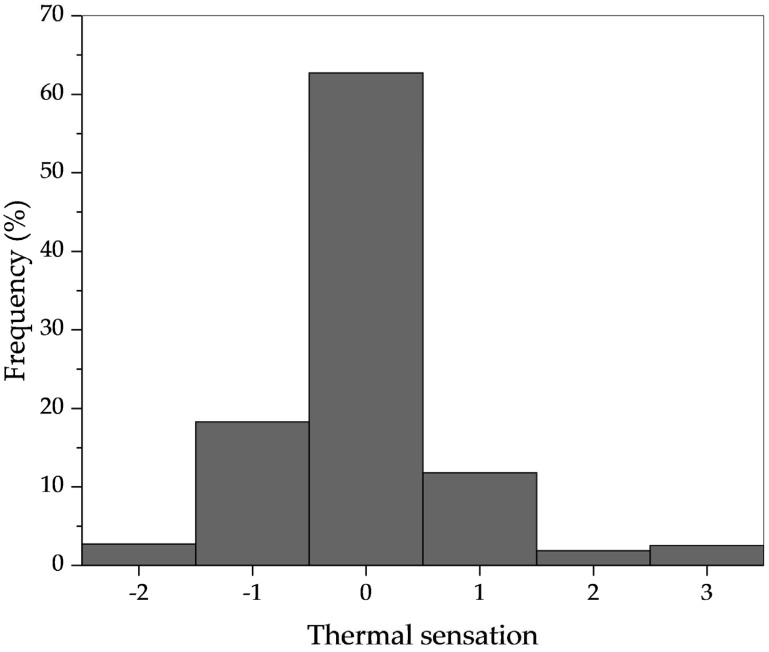
Distribution of thermal sensation vote.

**Figure 6:**
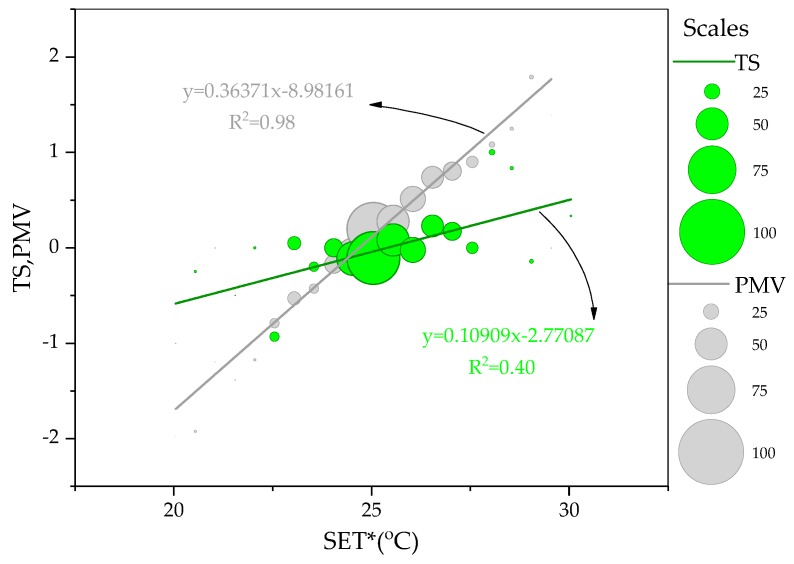
Comparison between TS and PMV against SET*.

Figure 5 presents the distribution of thermal sensation. The neutral thermal sensation was the most popular and more than 90% thermal sensation was located in the typical comfort zone (−1.0 to 1.0). Figure 6 compares the actual thermal sensation and PMV against indoor SET*. Letting the thermal sensation equal to zero, the neutral temperature was determined as 24.7 °C in SET*.

### 3.3. Thermal Acceptability

**Figure 7:**
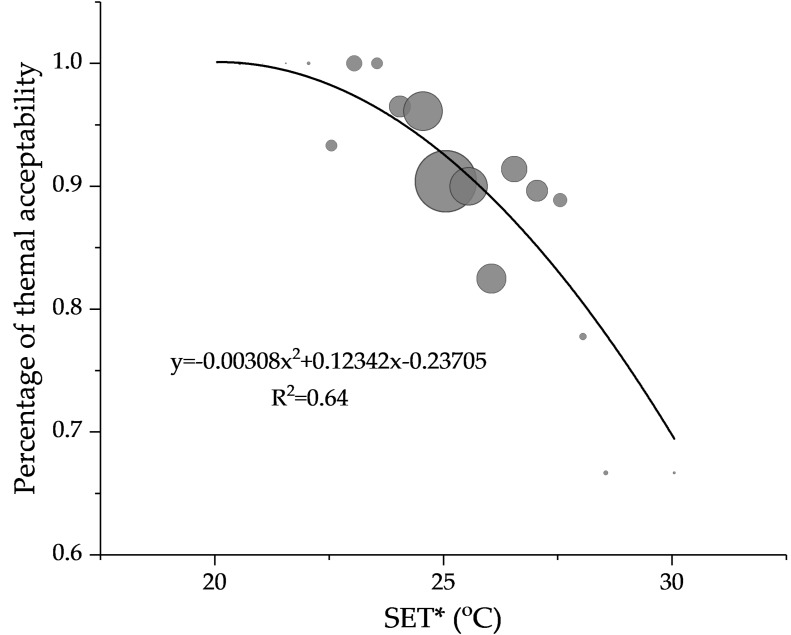
Determination of thermal acceptability in SET*.

To determine the acceptable temperature range, the relationship between acceptable percentage and indoor temperature was determined and the 80% acceptable temperature range [31] was analyzed. Since the investigated buildings were located in the HSCW climate zone, both the air temperature and humidity were high in summer. Occupants often use high-speed fans to maintain thermal comfort. It was necessary to consider the effect of humidity and air speed on thermal acceptability. Therefore, the SET* was adopted to evaluate the acceptable temperature. The data was divided into bins by SET* with an increment of 0.5 °C, and then the relationship between acceptable percentage and SET* was analyzed by using regression analysis weighted by sample size. The scatters show a tendency that the acceptable percentage changes with SET* as a second-order polynomial function (Figure 7). Therefore, second-order polynomial function was determined for the relationship. The acceptable percentage was inversely proportional to the SET* and decreased rapidly. The regression equation is listed as follows:

y = −0.00308x^2^ + 0.12342x − 0.23705, *R*^2^ = 0.64 (3)

Through regression, the upper limit for 80% acceptability is determined as 28.2 °C in SET*. Compared to the result derived from chamber experiment in Chongqing [38], with a similar climate to Changsha, the upper limit for 80% acceptability was 1.7 °C lower. This indicated that people in actual thermal environments accept a lower upper limit temperature.

### 3.4. Overall Thermal Comfort and Physiological Responses

The results of physiological measurements are presented in Table 4. The mean *T_finger_*, *T_wrist_*, *T_hand_* and *T_forearm_*, were 33.4 °C, 33.9 °C, 33.7 °C and 33.7 °C, respectively. The corresponding mean thermal sensation was −0.01.

**Figure 8:**
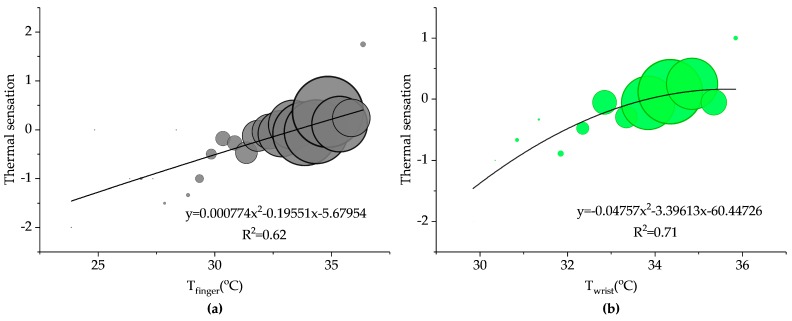

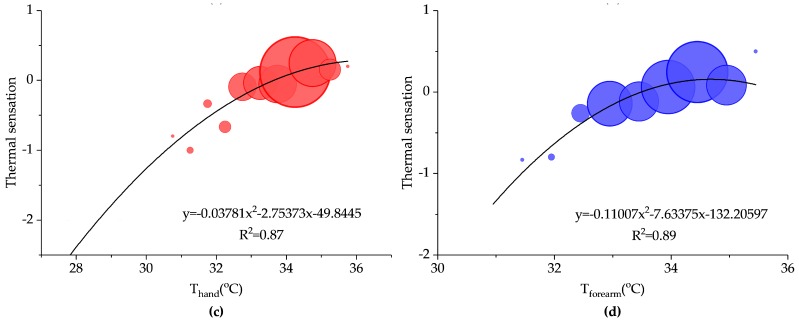
Relationships between the upper extremity skin temperatures and thermal sensation: (**a**) Thermal sensation vs. *T_finger_* (**b**) Thermal sensation vs. *T_wrist_* (**c**) Thermal sensation vs. *T_hand_* (**d**) Thermal sensation vs. *T_forearm_*.

The data was divided into bins by upper extremity skin temperatures with an increment of 0.5 °C, and then the relationships between different parameters were analyzed by using regression analysis weighted by sample size. The thermal sensation values were plotted against the upper extremity skin temperatures (Figure 8). To accurately indicate the relationships, second-order polynomial functions relating upper extremity skin temperatures and thermal sensation were established for the investigated occupants. To indicate the merit of the proposed methods on a more definite and tangible basis, statistical analysis comparisons between upper extremity skin temperatures evaluation approaches were conducted by conventional statistical indicator (i.e., *R*^2^). The greater absolute value of determination coefficient (*R*^2^) represents higher correlation relationship between the upper extremity skin temperatures and the actual thermal sensation. Therefore, the absolute value of *R*^2^ determined the order of priority of proposed approaches in thermal sensation prediction. The regression equations are listed as follows:

TS vs. *T_finger_*: y = 0.000774x^2^ − 0.19551x − 5.67954, *R*^2^ = 0.62, (4)

TS vs. *T_wrist_*: y = −0.04757x^2^ − 3.39613x − 60.44726, *R*^2^ = 0.71, (5)

TS vs. *T_hand_*: y = −0.03781x^2^ − 2.75373x − 49.8445, *R*^2^ = 0.87, (6)

TS vs. *T_forearm_*: y = −0.11007x^2^ − 7.63375x − 132.20597, *R*^2^ = 0.89, (7)

As shown in Figure 8, each upper extremity skin temperature is positively correlated with thermal sensation. Figure 8a shows the relationship between *T_finger_* and thermal sensation. A majority of points fell along the oblique line, and the determination coefficient was 0.62. Figure 8b–d present the relationships of *T_wrist_*, *T_hand_* and *T_forearm_*, respectively, in aspect of thermal sensation. The wrist and hand skin temperatures had determination coefficient of 0.71 and 0.87, respectively. The skin temperature of forearm had the highest determination coefficient of thermal sensation (*R*^2^ = 0.89), which implied that the *T_forearm_* model outperformed other models. The high determination coefficient indicated that thermal sensation could be well predicted by upper extremity skin temperatures. Also, the related neutral skin temperature (*NST*), namely the temperature corresponding to neutral thermal sensation, for each upper extremity skin was determined. The *NST_finger_*, *NST_hand_*, *NST_wrist_* and *NST_forearm_* were 33.5 °C, 33.6 °C, 33.8 °C and 33.5 °C, respectively. *NST* was close to the actual mean upper extremity skin temperatures (*T_finger_*: 33.4 °C, *T_wrist_*: 33.9 °C, *T_hand_*: 33.7 °C, *T_forearm_*: 33.7 °C), and the relative actual mean thermal sensation (−0.01) was almost equal to the neutral thermal sensation.

**Figure 9:**
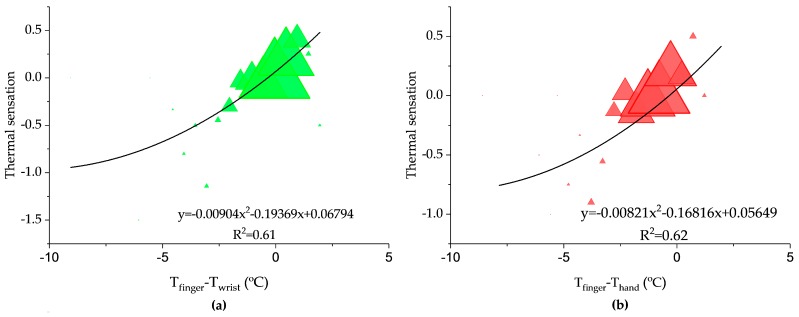

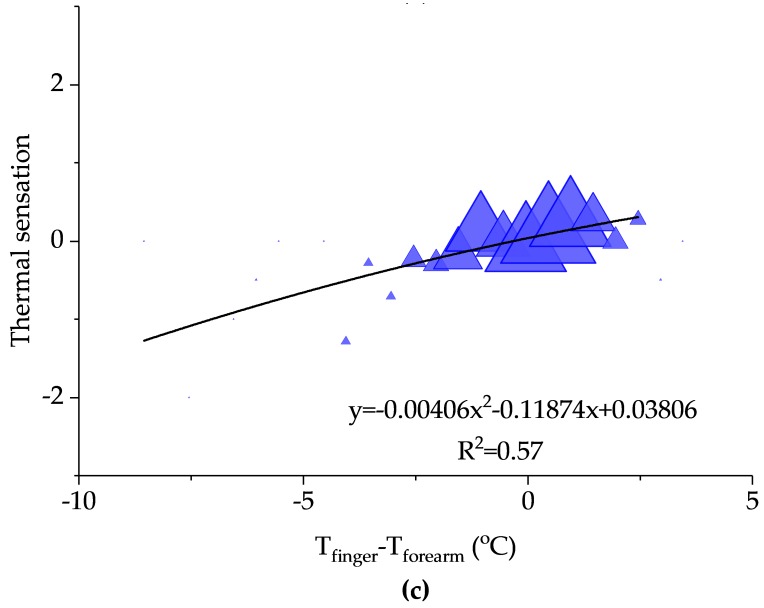
Relationships between the gradients of extremity skin temperatures and thermal sensation: (**a**) Thermal sensation vs. *T_finger-wrist_* (**b**) Thermal sensation vs. *T_finger-hand_* (**c**) Thermal sensation vs. *T_finger-forearm_*.

Rubinstein [39] demonstrated that the skin temperature gradient was well correlated with total fingertip blood flow and concluded that the skin temperature gradient was accurate measurement of thermoregulatory peripheral vasoconstriction. Therefore, apart from the evaluation of individual upper extremity skin temperatures, the relationships between the gradients of upper extremity skin temperatures (*GST_S_*) and thermal sensation were also determined (Figure 9). The determination coefficient for the *GST_S_* (between finger and wrist, hand and forearm) were 0.61, 0.62, and 0.57, respectively. The neutral gradients of upper extremity skin temperatures (*NGST_S_*) (*NGST_finger-wrist_*: 0.3 °C, *NGST_finger-hand_*: 0.3 °C, *NGST_finger-forearm_*: 0.3 °C), namely the temperature gradients corresponding to neutral thermal sensation, were close to 0. On the neutral-to-cool side, the gradients between finger and wrist, hand and forearm ranged from −3.5 to 0.3 °C, −4.2 to −0.3 °C and −4.0 to −0.3 °C, respectively. Both gradients between finger and wrist and hand showed a diminishing trend. It was believed that this phenomenon was the evidence of body thermoregulation to maintain thermal neutrality by vasodilatation and vasoconstriction in the blood vessels of the extremity [40]. Figure 9c shows that in warm condition, the finger-to-forearm gradient was consistently positive, because some occupants’ forearm started to sweat in warm condition.

### 3.5. Relationships between Thermal Environment and Upper Extremity Skin Temperatures

**Figure 10:**
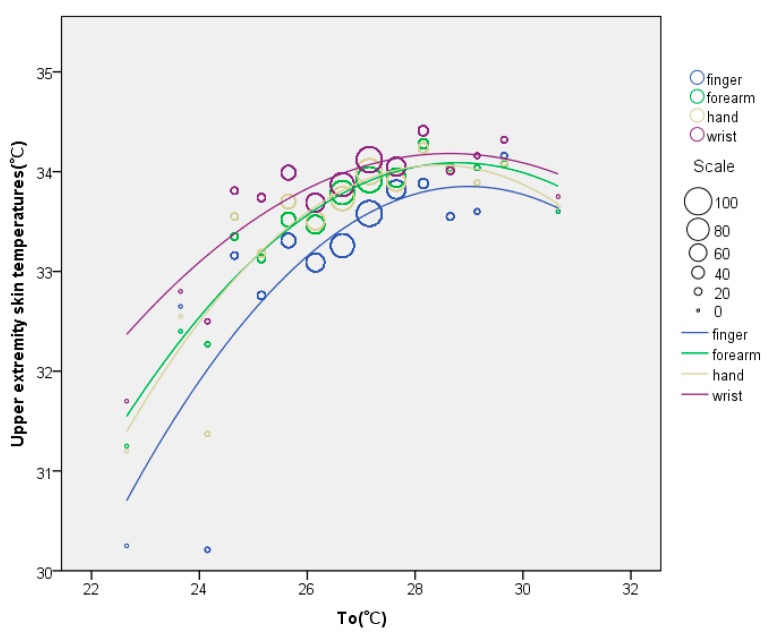
Relationships between *T_o_* and upper extremity skin temperatures.

To illustrate the relationships between the thermal environment and upper extremity skin temperatures, the upper extremity skin temperatures were plotted against the operative temperature (*T_o_*). The data was divided into bins by operative temperature with an increment of 0.5 °C, and then the relationships between different parameters were analyzed by using regression analysis weighted by sample size. As shown in Figure 10, second-order polynomial functions were established for upper extremity skin temperatures and *T_o_*. The upper extremity skin temperatures were proportional to *T_o_*. The related regression equations are presented as follows:

*T_o_* vs. *T_finger_*: y = −0.07836x^2^ − 4.54196x − 31.96692, *R*^2^ = 0.65, (8)

*T_o_* vs. *T_wrist_*: y = −0.05037x^2^ + 2.88556x − 7.14874, *R*^2^ = 0.63, (9)

*T_o_* vs. *T_hand_*: y = 0.08209x^2^ + 4.56489x − 30.81818, *R*^2^ = 0.71, (10)

*T_o_* vs. *T_forearm_*: y = −0.0676x^2^ + 3.89062x − 21.89523, *R*^2^ = 0.88, (11)

The *T_finger_* was the most widely distributed and the range of *T_forearm_* was smallest. An explanation for this difference could be that some people simply have a tendency to have cool fingers whereas others normally have warmer fingers. It was clear that *T_wrist_* was always higher than *T_hand_*, *T_forearm_* and *T_finger_*, and *T_finger_* was the lowest. According to the regression lines, *GST_S_* between finger and wrist, hand and forearm were maximized when *T_o_* was 22.5 °C. When *T_o_* ranged from 22.5 °C to 28 °C, the upper extremity skin temperatures became higher and *GST_S_* between finger and wrist, hand and forearm became smaller. When *T_o_* was larger than 28 °C, the upper extremity skin temperatures were almost constant and *GST_S_* between finger and wrist, hand and forearm were less than 0.5 °C. Moreover, the upper limits of upper extremity skin temperatures were found at approximately 34 °C, above which thermoregulation would be achieved by sweating [28].

### 3.6. Sex Differences

**Figure 11:**
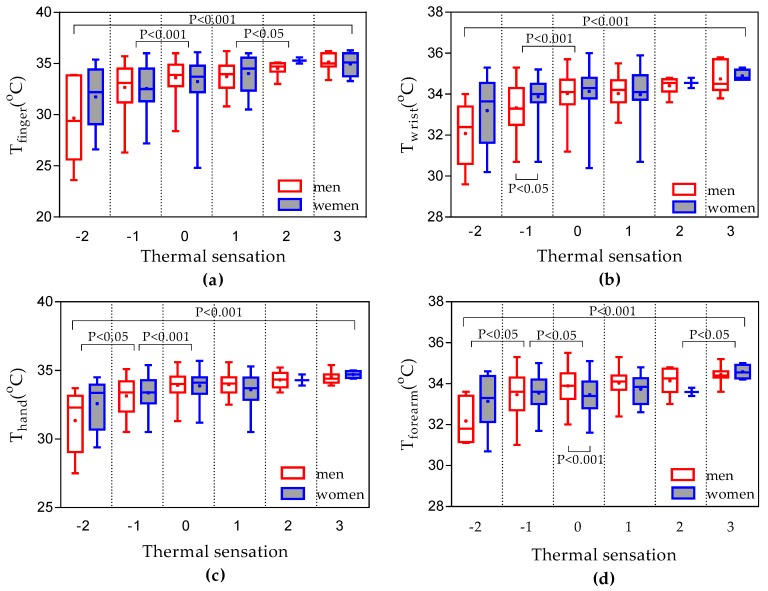
Upper extremity skin temperatures results: (**a**) sex difference in *T_finger_* (**b**) sex difference in *T_wrist_* (**c**) sex difference in *T_hand_* (**d**) sex difference in *T_forearm_.*

To explore whether there are significant differences in the upper extremity skin temperatures between women and men, the upper extremity skin temperatures were analyzed statistically. Figure 11 shows the upper extremity skin temperatures of different thermal sensation. Mann-Whitney test determined that there were no significant differences in upper extremity skin temperatures between men and women except *T_forearm_* (Table 5). In terms of each single thermal sensation group, men had significantly higher *T_forearm_* in neutral thermal sensation (*p* < 0.001). Women had significantly higher *T_wrist_* in cool thermal sensation (*p* < 0.05). In contrast, Humphreys [27] found a significant tendency for women to have cooler fingers than men during office work in the UK (women 31.1 °C, men 32.8 °C). This trend was not apparent in the results shown in Table 5, which similarly indicated that gender does not play a certain role in the finger temperatures.

## 4. Discussion

### 4.1. Predicted Mean Vote vs. Actual Thermal Sensation

To illustrate relationships of thermal sensation and PMV against SET*, linear regression was applied. Compared with PMV, the gentle gradient of actual thermal sensation suggested occupants were less sensitive to the environment and were able to adapt themselves to their current thermal environment. In detail, the PMV overestimated thermal sensation in warm conditions and underestimated the thermal sensation in cool conditions. In cool conditions, the discrepancies may be caused by using split air-conditioner and “cold-addiction” of occupants. In warm conditions, the discrepancies implied that various thermal behaviors adopted by occupants could make them comfortable at high temperature. The similar discrepancy between the actual thermal sensation and PMV could be found in other field studies [41,42,43,44,45,46], especially in the naturally ventilated and mixed-mode buildings, where the expectancy factor significantly influenced the occupants’ actual thermal sensation.

### 4.2. Possible Thermal Comfort Indicators

To assess the potential physiological indicators of thermal comfort, the relationships between upper extremity skin temperatures and thermal sensation were determined using second-order polynomial functions. Also, the relationships between *GST_S_* and thermal sensation were explored. The prediction of overall thermal sensation based on *T_forearm_* was higher (*R*^2^ = 0.89) than other upper extremity skin temperatures. Therefore, the *T_forearm_* evaluation model outperforms other upper extremity skin temperatures evaluation models. Moreover, a Kruskal-Wallis test showed that thermal sensation had a significant impact on upper extremity skin temperatures (Figure 11).

On the other hand, upper extremity skin temperatures changed significantly with *T_o_*, and increased with *T_o_* up to about 34 °C. This was a thermal stresses zone when the cutaneous temperature became larger than 34 °C, because the blood flow would no longer increase and sweat generation was required to control body temperature. Finger temperatures varied between individuals because their thermoregulatory set points differed. That was probably part of the reason that occupants showed such a large variation in finger temperature for the same sensation or operative temperature.

### 4.3. Limitation and Future Challenges

Although this study has determined the advantages of the upper extremity skin temperatures in evaluating thermal sensation, it was performed in the office buildings. Future research is required to focus on more evaluation of the suitability of upper extremity skin temperatures in the context of thermal comfort in different building types and places. Also, the upper extremity skin temperatures and other variables can be incorporated with each other to obtain higher precision and accuracy in future work. More combinations of physiological variables should be analyzed to develop the most influential set of parameters in this field.

## 5. Conclusions

An accurate thermal comfort assessment model is of great importance in improving the indoor thermal environment and reducing building energy consumption. In this study, upper extremity skin temperatures (i.e., finger, wrist, hand and forearm) were proposed as indicators of thermal comfort. Survey and measurements were conducted to investigate what potential relationships exist between the upper extremity skin temperatures and the thermal sensation and how the thermal environment was experienced by the people. The main conclusions are as follows:

(1) The neutral temperature is 24.7 °C and the upper limit for 80% acceptability is 28.2 °C in SET*.
(2) The high determination coefficient indicates that upper extremity skin temperatures may become potential physiological variables to evaluate thermal comfort of human beings. The forearm has the strongest relationship among the upper extremity.
(3) The relationships between thermal sensation and the gradients between the finger and the rest upper extremity skin temperatures are also established through second-order polynomial functions. It is clear that when the upper extremities temperature gradients are zero, the corresponding thermal sensations are almost equal to the neutral thermal sensation. When people feel cold, there are negative gradients. The positive gradients imply warm sensation.
(4) There are no significant differences in upper extremity skin temperatures between men and women except *T_forearm_*.

This study not only provides fundamental data for understanding the thermal environment but also presents the corresponding thermal comfort in the HSCW zone of China. Moreover, this study indicates that the upper extremity skin temperatures may become potential physiological variables to evaluate the thermal comfort of human. Thermal comfort of human may also be reflected by upper extremity skin temperature gradients, which may become a promising way to assess thermal comfort in objective physiological mechanisms.

## Figures and Tables

**Table 1:** The anthropometric information of the investigated occupants.

Gender	Number	Age	Height (cm)	Weight (kg)	BMI ^a^ (kg/m^2^)	Surface Area (m^2^)
Male	298	26.0 ± 5.1 ^b^	172.1 ± 5.0	65.0 ± 7.9	21.9 ± 2.3	1.76 ± 0.11
Female	132	26.4 ± 6.2	160.9 ± 4.0	50.1 ± 5.2	19.3 ± 1.7	1.50 ± 0.08
All	430	26.1 ± 5.5	168.7 ± 7.0	60.4 ± 10.0	21.1 ± 2.4	1.68 ± 0.16

^a^ Body mass index [32], BMI = weight/height, normally between 18 and 25 kg/m^2^; ^b^ Standard deviation.

**Table 2:** Metrological properties of related instruments.

Parameters	Instruments	Range	Accuracy	Resolution
Air temperature	Thermo recorder TR-72U	−10–60 °C	±0.3 °C	0.1 °C
Relative humidity	Thermo recorder TR-72U	10–95% RH	±5% RH	1% RH
Globe temperature	HEART INDEX CHECKER 8778	0–50 °C	±0.6 °C	0.1 °C
	WBGT-2009	0–80 °C	±0.6 °C	0.1 °C
Air velocity	Testo 425	0–20 m/s	±0.03 m/s	0.01 m/s
Skin temperature	Testo 905-T2	−50–350 °C	±1.0 °C	0.1 °C

**Table 3:** The distribution of thermal parameters.

Variables	*T_a_* (°C)	*T_g_* (°C)	*RH* (%)	*V_a_* (m/s)	*T_o_* (°C)	*T_r_* (°C)	SET* (°C)	INSUAL (clo)
25th Percentile	26.2	26.2	49.3	0.08	26.2	26.2	24.5	0.31
75th Percentile	27.6	27.6	61	0.2	27.6	27.6	26.3	0.4
Skewness	−6.63	−7.36	−0.45	2.84	−7.28	−7.47	−0.89	0.71
Kurtosis	92.41	107.4	3.9	11.22	105.7	110.5	31.98	3.22

Note: *T_a_*, Mean air temperature; *T_g_*, Globe temperature; *RH*, Relative humidity; *V_a_*, Mean air velocity; *T_o_*, Operative temperature; *T_r_*, Mean radiant temperature; SET*, Standard effective temperature; INSUAL, Clothing insulation.

**Table 4:** Results of physiological measurements.

Variables	*T_finger_* (°C)			*T_wrist_* (°C)			*T_hand_* (°C)			*T_forearm_* (°C)		
	M	F	M+F	M	F	M+F	M	F	M+F	M	F	M+F
Mean	33.5	33.2	33.4	33.9	33.5	33.7	33.9	34.0	33.9	33.8	33.7	33.7
SD	1.8	2.2	1.9	0.9	0.8	0.9	1.0	1.1	1.0	1.0	1.2	1.1
Maxi	36.2	36.3	36.3	35.5	35.1	35.5	35.8	36.3	36.0	35.6	35.7	35.7
Mini	23.6	24.8	23.6	31.0	30.7	30.7	29.6	30.2	29.6	27.5	29.4	27.5

Note: SD, Standard deviation; Maxi, Maximum; Mini, Minimum; *T_finger_*, Finger skin temperature; *T_forearm_*, Forearm skin temperature; *T_wrist_*, Wrist skin temperature; *T_hand_*, Hand skin temperature.

**Table 5:** Mean upper extremity skin temperatures of investigated occupants.

TS	−2	−1	0	1	2	3	All
*T_finger_*							
M	29.7 ± 4.4	32.7 ± 2.2	33.6 ± 1.5	33.7 ± 1.3	34.5 ± 0.4	35.1 ± 1.0	33.5 ± 1.8
F	31.7 ± 3.2	32.6 ± 2.1	33.3 ± 2.1	34.0 ± 1.8	35.3 ± 0.4	35.0 ± 1.2	33.2 ± 2.2
M+F	30.8 ± 3.7	32.6 ± 2.1	33.5 ± 1.7	33.8 ± 1.5	34.7 ± 0.8	35.1 ± 1.0	33.4 ± 1.9
*T_wrist_*							
M	32.1 ± 1.6	33.3 ± 1.2 *	34.0 ± 0.9	34.0 ± 0.8	34.4 ± 0.4	34.7 ± 0.8	33.9 ± 1.0
F	33.2 ± 1.8	33.9 ± 1.0 *	34.1 ± 1.0	34.0 ± 1.3	34.6 ± 0.4	34.9 ± 0.3	34.0 ± 1.1
M+F	32.7 ± 1.8	33.6 ± 1.7	34.1 ± 0.9	34.0 ± 1.0	34.6 ± 0.4	34.8 ± 0.6	33.9 ± 1.0
*T_hand_*							
M	31.3 ± 2.4	33.2 ± 1.3	33.9 ± 0.8	34.0 ± 0.8	34.3 ± 0.6	34.5 ± 0.5	33.8 ± 1.0
F	32.6 ± 1.9	33.4 ± 1.3	33.9 ± 1.0	33.6 ± 1.3	34.3 ± 0.6	34.7 ± 0.3	33.7 ± 1.2
M+F	32.0 ± 2.2	33.2 ± 1.3	33.9 ± 0.9	33.9 ± 0.9	34.3 ± 0.6	34.6 ± 0.4	33.7 ± 1.1
*T_forearm_*							
M	32.2 ± 1.2	33.5 ± 1.1	33.89 ± 0.78 ***	34.0 ± 0.6	34.1 ± 0.7	34.4 ± 0.5	33.9 ± 0.9 ***
F	33.1 ± 1.4	33.6 ± 0.8	33.47 ± 0.80 ***	33.7 ± 0.7	33.6 ± 0.3	34.6 ± 0.4	33.5 ± 0.8 ***
M+F	32.7 ± 1.3	33.5 ± 1.0	33.77 ± 0.81	34.0 ± 0.7	34.0 ± 0.6	34.5 ± 0.4	33.7 ± 0.9

Note: Data is presented as mean ± SD. M = men, F = women; All, All occupants; *T_finger_*, Finger skin temperature; *T_forearm_*, Forearm skin temperature; *T_wrist_*, Wrist skin temperature; *T_hand_*, Hand skin temperature; * *p* < 0.05, difference between women and men; *** *p* < 0.001, difference between women and men.
